# Traumatic anterior tibiofemoral dislocation of mobile-bearing total knee arthroplasty: Two cases

**DOI:** 10.1016/j.tcr.2025.101144

**Published:** 2025-02-17

**Authors:** Carlijn Schoutens, Peter A. Nolte, Arthur van Noort

**Affiliations:** a Department of Orthopaedic Surgery, Spaarne Gasthuis, Spaarnepoort 1, 2134 TM Hoofddorp, the Netherlands; b Dept Oral Cell Biology, Academic Centre for Dentistry (ACTA), University of Amsterdam and Vrije Universiteit Amsterdam, Gustav Mahlerlaan 3004, 1081 LA Amsterdam, the Netherlands; c (EURECA) Erasmus Medisch Centrum Rotterdam, Dr. Molewaterplein 40, 3015 GD Rotterdam, the Netherlands

**Keywords:** Anterior dislocation, Knee dislocation, Total knee arthroplasty, Risk factor, Neurovascular injury, Mobile bearing

## Abstract

**Background:**

Anterior tibiofemoral dislocation is a severe complication of a total knee arthroplasty. It is rare, and it is distinctly different from bearing spinout. Most tibiofemoral dislocations are posterior. Anterior dislocation has previously been described in various prosthesis designs, but not in mobile-bearing prostheses. We present two cases and provide recommendations for the management of this rare and severe injury.

**Case description:**

Two cases of complete anterior tibiofemoral dislocation were brought on by trauma, fifteen and eight years after initial implantation of mobile-bearing total knee arthroplasties in 71-year-old and 73-year-old female patients. One was managed with closed reduction and made a full recovery. In the other, closed reduction failed, open reduction was performed, and there was a need for revision surgery for instability after her initial recovery. There were no neurovascular complications. Follow-up was 23 and 14 months respectively.

**Conclusion:**

Anterior tibiofemoral dislocation is a severe injury with a risk of concomitant complications. Early management should include prompt reduction, serial neurovascular exams and CT angiography for all cases. Late management should include assessment of joint stability.

## Highlights

• Low-energy trauma can cause complete tibiofemoral dislocation of total knee arthroplasty.
• Complete dislocation in mobile-bearing knee arthroplasty is different from bearing spinout.
• Anterior tibiofemoral dislocation requires prompt reduction.
• Clinical assessment and imaging are indicated to rule out concomitant neurological or vascular injury.
• Long term sequelae may include persistent instability and the need for revision surgery.

## Introduction

We present two cases of complete anterior tibiofemoral dislocation fifteen and eight years after primary rotating-platform Total Knee Arthroplasty (TKA) (LCS Complete™ Rotating Platform cementless; DePuy, Warsaw, IN). Complete anterior dislocation has not previously been described in mobile-bearing TKA designs, and is also rare in other TKA designs. The injury carries a risk of severe vascular and neurologic complications and should therefore be regarded differently than the more common dislocation of the insert in mobile-bearing arthroplasties. No established treatment guideline exists. It is imperative to share widely the scarce experiences with this uncommon injury to inform the optimal management of future cases.

## Cases

Both patients whose cases are described below gave informed consent for the use of their medical information and images in this publication.

### Case 1

A 71-year-old Caucasian woman presented to the Emergency Department after a fall from stance onto the floor in the domestic setting, in which hyperextension of her left prosthetic knee joint had probably occurred. She reported immediate severe pain and deformity and inability to bear weight. A LCS Complete™ cementless Rotating Platform TKA had been implanted fifteen years prior for symptomatic lateral osteoarthritis. The surgery and recovery had been uncomplicated, the prosthetic joint had had excellent functionality and the patient was highly satisfied. At routine physical examination seven months prior to her fall, the range of motion was from 0^0^ to 130^0^ with no instability. Conventional radiographic images showed no signs of complications. Her past medical history further included a total knee arthroplasty on the contralateral side five years ago for osteoarthritis. Her Body Mass Index (BMI) was 29 kg/m^2^. Her Karnofsky Performance Scale score was >90 %.

**Figs. 1 and 2:**
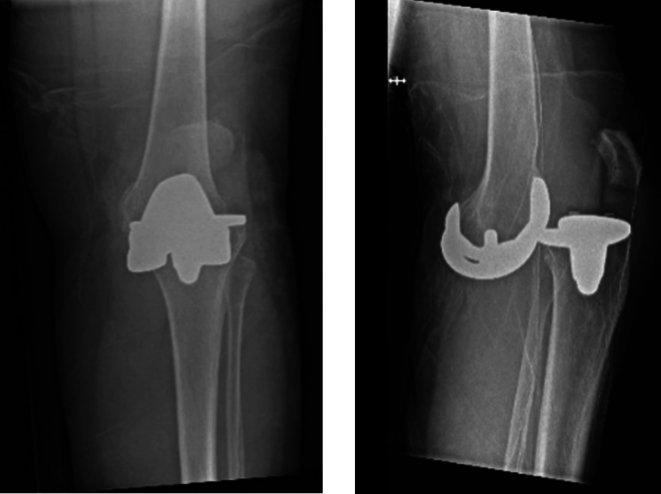
Case 1: conventional radiography images at time of presentation.

The patient was in severe pain and there was a notable deformity of her left knee. Physical examination of the prosthetic joint was not possible due to pain. Neurological examination showed normal sensitivity without numbness or tingling. Muscle strength for all foot and toe movements was symmetric. The foot was warm and pink. Strong peripheral pulses were palpable. Dislocation of the TKA or a periprosthetic fracture with displacement were suspected. Radiographic images confirmed the diagnosis of complete anterior TKA dislocation (Figs. 1, 2). There was also a nondisplaced fracture of the medial femoral condyle.

It was decided to attempt a closed reduction and proceed with open reduction if this failed. A senior orthopedic surgery resident under supervision of a senior orthopedic surgeon performed the procedure. The joint was successfully reduced with axial traction and minimal hyperextension, as was clearly felt by the physician and confirmed radiographically. The postprocedural radiograph showed normal alignment and there were no signs of new complications. The neurovascular status remained unchanged. Computed tomography (CT) imaging was done to assess the osseous injury more closely and check for vascular complications such as intimal dissection. There were no signs of vascular injury or thrombosis, although the image quality was poor in the popliteal area of interest due to scatter from the TKA.

**Figs. 3 and 4:**
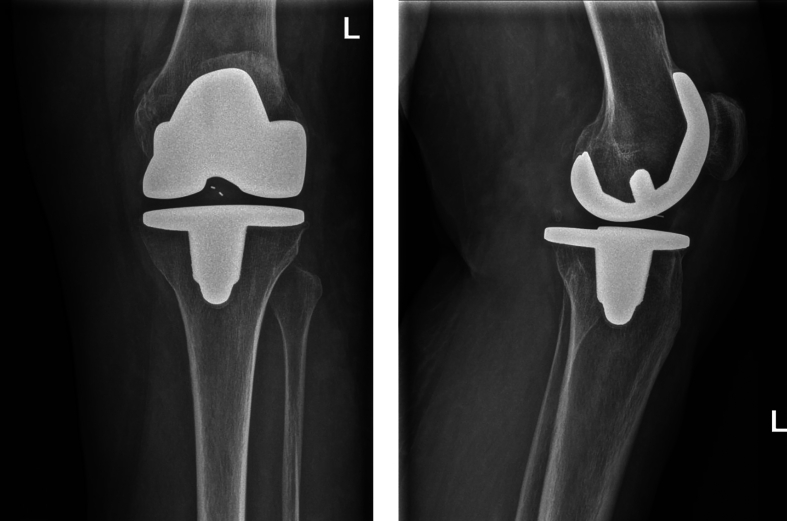
Case 1: conventional radiography images during follow-up.

The patient was admitted to the hospital for pain management and neurovascular status monitoring, which remained normal. A brace was applied and locked in extension. On the second day, the brace was adjusted to allow flexion from 10 to 45 degrees and she was cleared for functional weight-bearing. She was discharged on the third day and instructed to wear the brace continuously and continue physiotherapy. Follow-up consultations took place through outpatient clinic visits with a senior orthopedic surgeon. The brace was gradually adjusted to allow for more mobility. Conventional radiographs showed ongoing healing of the fracture (Figs. 3, 4). At the three-month visit, there was subsidence of all pain, range of motion of 115–4-0 and mediolateral and anteroposterior stability equal to the preoperative assessment. She was instructed to gradually reduce her usage of the brace. At the eight-month visit, the ROM was 130–0-5 and the patient was fully satisfied and free of pain. The fracture was consolidated. There was no recurrence at 23-months follow up and no further treatment or follow-up was deemed necessary.

### Case 2

A 73-year old Caucasian woman presented to the Emergency Room after slipping on an oil spill and falling directly onto her left knee. She was in severe pain, could not bear weight, and had a deformed left knee joint. A left LSC Complete™ cementless Rotating Platform TKA had been implanted 8 years prior for symptomatic osteoarthritis in an uncomplicated procedure. In subsequent years she had multiple clinic visits for ongoing symptoms: persistent feelings of instability and an intermittent locking sensation. Physical exam one year prior to this incident was positive for minimal swelling and grade 1 collateral ligamentous laxity; ROM was 120–0-10. Conventional radiography of the knee, lower spine and pelvis had not revealed abnormalities and bone scans had been negative for signs of loosening. A CT scan was positive for retropatellar cartilage loss. Insert replacement had been discussed with the patient. The decision was made to first attempt treatment with a brace. On the contralateral side, where she had similar discomfort despite a different TKA design (Genesis; Smith & Nephew, Memphis, TN) implanted by another surgeon in another hospital ten years earlier, she had had satisfactory results with insert replacement and patellar prosthesis implantation three years earlier. Her past medical history further included an incidental finding of old cerebral infarction and obstructive sleep apnea syndrome. Her BMI was 26 kg/m^2^.

**Figs. 5 and 6:**
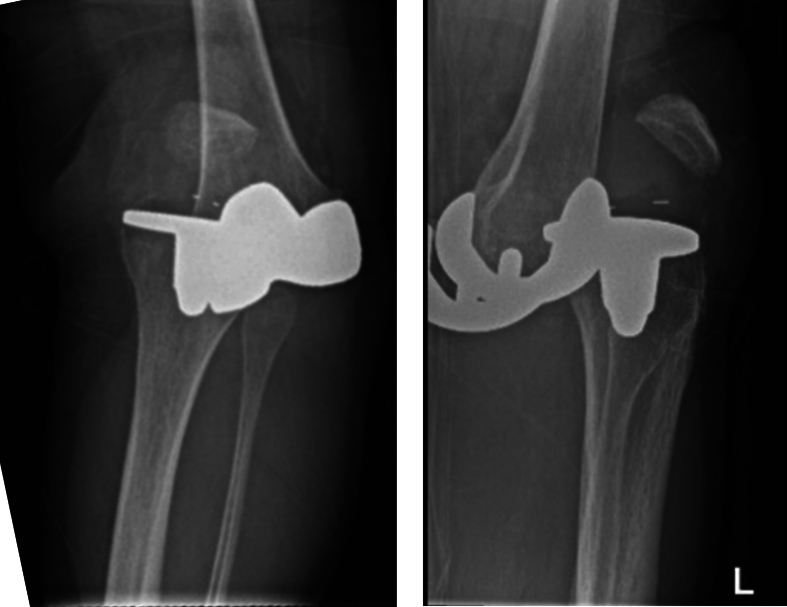
Case 2: conventional radiography images at time of presentation.

Upon presentation, there was a deformity. Distal sensibility and strength were normal; peripheral pulses were strong and the foot was warm. Radiographic images confirmed the suspected diagnosis of TKA dislocation (Figs. 5, 6).

**Figs. 7 and 8:**
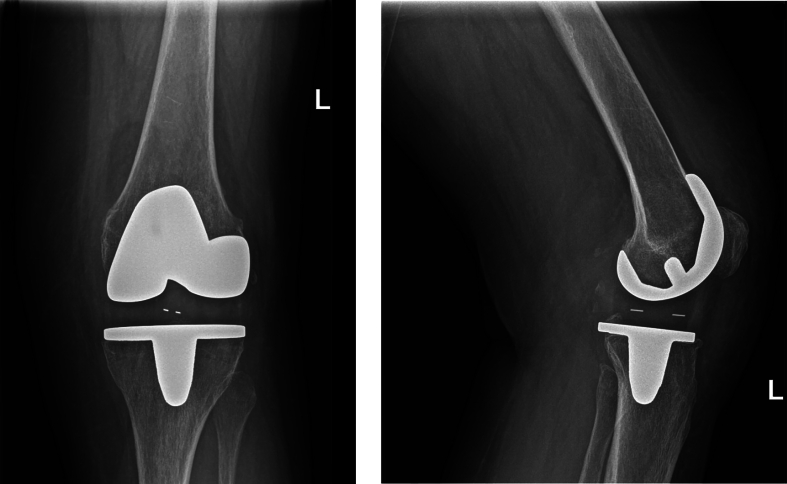
Case 2: conventional radiography images after revision surgery.

Closed reduction was attempted by a senior orthopedic surgeon. Repeat X-rays showed partial reduction: the prosthetic joint was realigned laterally but not medially. It was decided to proceed with open reduction. Preoperative testing under anesthesia was positive for anteroposterior ligamentous laxity as well as medial and lateral grade 2 laxity and a hard stop in both directions. During the surgery, the original insert was found in its correct position and it was not replaced. A postoperative CT angiogram showed no evidence of vascular injury and no osseous injury. The joint was immobilized in extension with a cast. She was admitted to the hospital for pain management and observation. There were no late clinical signs of vascular or neurological dysfunction. She was discharged on the second day. Postoperative treatment was continuation of cast immobilization for 6 weeks. At the six-week follow-up visit, there was laxity with hyperextension, leaving her at risk for a recurrence. Therefore, a locking brace was given and set to 90–5-0 degrees for 24 h per day, and physiotherapy was initiated. Long-term follow-up after twelve months showed persistent symptomatic instability. A shared decision was made to plan revision surgery. The insert, which showed some signs of wear, was explanted and replaced by a thicker insert. The knee was stable during intra-operative testing; no further revision such as to a semi constrained design was necessary. Postoperative conventional radiographic images showed no signs of complications (Figs. 7, 8). She had an uncomplicated recovery with a stable knee at 6-week follow-up.

## Discussion

### Epidemiology; lack of known similar cases

To our knowledge, there has not been a previous case of anterior tibiofemoral dislocation of a mobile-bearing TKA described in the literature. In other TKA designs, anterior dislocations have been reported. The majority are posteriorward. A systematic review by Rouquette et al. on tibiofemoral dislocations reported 11 % anterior. [1] On anterior tibiofemoral dislocations, Almoguera-Martinez et al. presented a case and reviewed fifteen more. Kim et al. reviewed the literature also, identifying one additional case and adding one more from experience. [2,3] Four additional cases were found by the authors of this study, to make a total of twenty-two before the patients in this paper. [[4], [5], [6], [7]] None of the previously described cases have occurred in mobile-bearing prostheses.

### General risk factors

Causes and contributing factors to the occurrence of complete anterior tibiofemoral TKA dislocation are not well-established. Other authors have pointed to the suspected risk factors of female gender, obesity, previous severe deformity, metabolic conditions such as rheumatoid arthritis and diabetes that lead to weakening of ligamentous joint stability, and conditions such as delirium that lead to an increased propensity for trauma. [[1], [2], [3]] Female gender is present in twenty of the twenty-two other known anterior tibiofemoral dislocation cases. Bigham et al. have shown that morbid obesity, defined as BMI ≥40 kg/m^2^, poses a greater risk for revision surgery due to all-direction tibiofemoral dislocation than non-morbid obesity does. [8] This may be because high BMI places larger demands on the prosthetic joint and necessitates greater exposure during the initial surgery. [6] Implant wear is an additional suspected risk factor. It has been inferred that anterior dislocation is associated with a greater degree of implant wear than posterior dislocation. This is supported by the observation that anterior dislocations tend to occur much later in the postoperative course than posterior dislocations. [1,3]

In terms of gender, presence of trauma, and time between implantation and dislocation, the cases in this report fall in line. In contrast to other cases, both cases in this report do not have known metabolic disorders besides osteoarthritis.

### Mechanical factors

The mechanisms of injury were low-energy trauma in both cases. It is consistent with the literature that this can be a sufficient trauma mechanism to cause dislocation [9,10] This is in contrast with dislocations of the native knee joint, which require higher-energy impact. [11] Inherent mechanical factors predisposing to dislocation of a TKA are those that lead to prosthetic joint instability. The concept of mobile-bearing prostheses aims to improve implant survival through its design. It is intended to reduce shear stress forces on the bone-implant interface through allowing more mobility at the level of the polyethylene insert. [12] This is known to come with a risk of complications, such as bearing spinout, that are not seen in fixed bearing prosthesis designs. Risk factors for bearing spinout are reduced thickness of the bearing due to wear, and soft tissue laxity. [13]. Flexion gap instability due to improper balancing is also a suspected risk factor for bearing spinout. [14] For anterior tibiofemoral dislocation in non-mobile-bearing designs, purported surgical risk factors are medial collateral ligament damage, posterior cruciate ligament damage, excessive tibial tilt, tibial component malrotation, femur component malposition, quadriceps atrophy, and patellar tendon rupture. [3] Other authors also point to severe polyethylene wear, valgus deformity, greater laxity in flexion than extension, and posterior capsule tear. [9,[15], [16], [17]] We hypothesize that true surgical error likely would lead to problems in the initial few years after implantation. In a review of all-direction tibiofemoral dislocations, it was found that 44 % dislocate in the initial 6 months and only 13 % dislocate >5 years after implantation. [1] Anterior dislocations more often are late dislocations. We hypothesize that in late dislocations, trauma becomes a more important risk factor. The traumatic mechanism in most cases is hyperextension with a rotational force. [3] In case 1 of this report, there were no known ligamentous or mechanical problems, but the medial condyle fracture that occurred with the trauma may have compromised its stability. In case 2, there was pretraumatic subjective laxity and insert revision was already being discussed with the patient for this reason. However, radiographic examination by X-ray and CT had not revealed a definitive cause. Both TKAs were implanted in the same hospital, but by different surgeons. In a cohort of LCS prostheses in this hospital, an extensive survival analysis has been carried out among 1289 implants in 1068 patients. [18] It found an all-cause 15-year survival rate of 96 % with functional outcomes similar to national reference values and good patient satisfaction. There were 49 revisions, two of which were due to insert spin-out and none of which were due to tibiofemoral dislocation.

### Complications and recommended treatment

As with complete dislocation of the native knee joint, there is a risk of severe complications with tibiofemoral prosthetic joint dislocation. Popliteal artery injury is likely: nine of the twenty-two previously known cases had tear, occlusion, dissection or transection of this artery. Seven had vascular bypass surgeries, with or without distal fasciotomies to prevent compartment syndrome from reperfusion injury. Four of those recovered successfully with no residual symptoms; one was stabilized but suffered permanent neurological damage. In two patients, amputation was ultimately necessary and in one of those, multi-organ failure led to the patient's death. [3,[5], [6], [7],^9^,^15^,^16^,^19^,^20^] Consequently, it is indicated in all patients after anterior tibiofemoral TKA dislocation to perform contrast-enhanced imaging of the popliteal artery by CT angiography.

Neurological damage after TKA dislocation is usually secondary due to ischemia from the vascular injury. [9,19] In one previous case, peroneal nerve palsy was described. [16] We recommend that early management include prompt reduction and neurological examination for all cases.

Long-term sequelae of tibiofemoral prosthetic joint dislocation include symptomatic instability. If open reduction is performed, we recommend that the need for an insert exchange is considered. Management after reduction should include external stabilization, such as by a brace or cast, to prevent early recurrence. Long-term management should include repeated assessments of range of motion and stability. It should be assessed, with consideration to the various risk factors discussed in this article, if there is a need for revision surgery to prevent recurrence or alleviate residual symptoms.

## Conclusion

Anterior tibiofemoral dislocation of a total knee arthroplasty is a rare and serious injury with a high risk of complications. Early management should include prompt reduction, serial neurovascular assessment and CT angiography for all cases.

## CRediT authorship contribution statement

**Carlijn Schoutens:** Writing – original draft, Validation, Methodology, Investigation, Formal analysis, Conceptualization. **Peter A. Nolte:** Writing – review & editing, Validation, Supervision, Methodology, Investigation, Formal analysis, Conceptualization. **Arthur van Noort:** Writing – review & editing, Validation, Supervision, Investigation, Formal analysis, Conceptualization.

## Funding

This research did not receive any specific grant from funding agencies in the public, commercial, or not-for-profit sectors.

## Declaration of competing interest

Arthur van Noort reports a relationship with Lima Italy that includes: consulting or advisory. The other authors declare that they have no known competing financial interests or personal relationships that could have appeared to influence the work reported in this paper.
